# Novel Selective Anticancer Effect of Epididymis-Derived Extracellular Vesicles Against HCC38 and MCF-7 Breast Cancer Cell Lines

**DOI:** 10.3390/ijms27093870

**Published:** 2026-04-27

**Authors:** Razi Zoabi, Zenab Ali Saleh, Elias Issaq, Etedal Morad, Reem Miari, Hanan Taha, Ahmad Kadriya, Abraham O. Samson, Mizied Falah

**Affiliations:** 1 Medical Research Institute, The Holy Family Hospital, Nazareth 16100, Israel; 2 Azrieli Faculty of Medicine, Bar-Ilan University, Safed 1311502, Israel; 3 Department of Urology, The Holy Family Hospital, Nazareth 16100, Israel; 4 Department of Pathology, The Holy Family Hospital, Nazareth 16100, Israel; etedal.m@hfhosp.org; 5 Exocure Therapeutics, Industrial Park, Sakhnin 308100, Israel

**Keywords:** extracellular vesicles, epididymis-derived EVs, breast cancer, apoptosis, p53

## Abstract

Prevalent cancers primarily include breast, lung and bronchus, prostate, and colorectal cancers. In contrast, cancer of the epididymis is very rare, and we propose that this tissue could carry inherent anticancer components, in particular, small extracellular vesicles (EVs) with antineoplastic properties. All cell types release extracellular vesicles (EVs) into their intercellular space, which act in the crosstalk required to achieve homeostasis. Among these, small EVs, which are membrane-bound vesicles with an average diameter of 30–200 nm, can transfer cell-specific cargo, such as lipids, proteins, DNA and RNA, which can be selectively received by neighboring or distant cells, and trigger specific cell processes, such as growth, division, or apoptosis. Here, we isolated small EVs from epididymis tissue, and examined their effect on morphology, viability, apoptosis, cell cycle phases, and certain gene and protein expression levels, particularly of the pro-apoptotic p53 protein, in HCC38 and MCF-7 breast cancer cell lines, as well as in a normal fibroblast cell line. The various analyses demonstrated effects on breast cancer cells but not on normal cells. Specifically, epididymis-derived EVs (Ep-EVs) selectively induced apoptosis and cell cycle arrest in cancer cells, while normal cells were unaffected. Moreover, the relative uptake of Ep-EVs in HCC38 and MCF-7 breast cancer cells was significant, indicating a direct association between vesicle internalization and the biological response. Taken together, these findings demonstrate a solid experimental foundation supporting the therapeutic potential of Ep-EVs in breast cancer, with promising implications for their development as a broader anticancer platform.

## 1. Introduction

Breast cancer is one of the most common malignancies worldwide and remains a leading cause of cancer-related mortality among women [1,2,3]. Despite significant advances in diagnosis and treatment strategies, some types of breast cancer remain difficult to cure. Historically, breast tumors have been treated with different strategies. Surgical management therapy has evolved from radical mastectomy to modified versions thereof, and more recently to breast-conserving surgery guided by locoregional approaches [4,5]. In addition, systemic chemotherapy is used to treat tumors with a poor prognosis [6]. Neoadjuvant chemotherapy is administered before surgery to reduce tumor size, improve surgical outcomes, and limit metastatic spread [7]. In contrast, adjuvant chemotherapy and radiotherapy are administered after surgery, to eliminate residual malignant cells and thereby reduce the risk of cancer recurrence [8]. Importantly, chemotherapy and radiotherapy are associated with severe side effects that compromise patients’ quality of life [9,10,11]. In addition, cancer cells often develop complex resistance mechanisms, enabling them to evade treatment. For example, breast tumors can develop resistance to tamoxifen, an antihormonal therapy that targets the estrogen receptor [12]. The cells achieve this by converting the toxic superoxide anion produced by tamoxifen into hydrogen peroxide, which is less toxic [12]. More recently, advances in immunotherapy have facilitated the development of personalized therapeutic strategies tailored to the molecular characteristics of patients [13]. For example, tumors that overexpress human epidermal growth factor receptor 2 (HER2) have been treated with trastuzumab, a recombinant monoclonal antibody that targets HER2 [14]. Yet, breast tumors can also develop resistance to immunotherapies, such as trastuzumab. Cancer cells accomplish this through the action of MUC4, a membrane glycoprotein that binds HER2, and increases phosphorylation of Tyr1248, thus activating the HER2 oncoprotein [15,16,17]. The adaptive strategies of breast tumors highlight the urgent need for new resistance-proof, low-toxicity therapeutic strategies [9,10,11].

Extracellular vesicles (EVs) are produced by cells that carry a cell-specific combination of molecular cargo, which can be taken up by neighboring cells via fusion or endocytosis [18,19,20]. EVs have emerged as promising therapeutic agents with potential anticancer properties. Notably, EVs secreted from mesenchymal stem cells (MSCs) derived from Wharton’s jelly connective tissue induce apoptosis in cervical cancer cells [21], and activate apoptosis in ovarian cancer cells [22]. Importantly, EVs derived from non-malignant tissues have been shown to exert tumor-suppressive effects, including the induction of apoptosis, inhibition of proliferation, and modulation of cell cycle checkpoints in cancer cells [21,22,23,24]. These effects are often mediated through EV-associated regulatory cargo influencing p53-dependent and p53-independent apoptotic pathways [25]. These findings indicate that EVs can carry diverse regulatory and pro-apoptotic cargo capable of suppressing tumor growth through multiple complementary mechanisms. As such, EVs are now recognized as key mediators of intercellular communication, capable of transferring bioactive proteins, lipids, and nucleic acids that directly modulate recipient cell fate [23].

The epididymis represents a particularly compelling candidate source of tumor-suppressive EVs. The epididymis is a tightly coiled tube located on the posterior surface of each testis. It is a highly specialized organ characterized by an exceptionally low incidence of malignant transformation compared with most somatic tissues [26]. This relative cancer resistance has been attributed to tightly regulated epithelial turnover, robust surveillance of DNA damage, controlled apoptotic signaling, and a unique immune-privileged microenvironment that limits chronic inflammation and oncogenic stress [26,27]. In addition, transcriptomic and functional studies have demonstrated that epididymal epithelial cells exhibit enhanced regulation of oxidative stress responses, p53-associated checkpoint pathways, and pro-apoptotic signaling cascades, all of which are central to tumor suppression [28,29]. Aside from its role in spermatozoa maturation and storage, the epididymis keeps spermatozoa viable and protected from invasive pathogens and autoimmune agents [26,28,30]. Epididymosomes are EVs derived from sperm fluid that have been studied in the context of sperm maturation and immune tolerance. EVs are known to transport diverse regulatory proteins, stress-response molecules, and microRNAs essential for maintaining cellular homeostasis [30,31]. Notably, epididymal EVs are secreted from pseudostratified epithelial cells, comprising four main cell types (i.e., principal, basal, clear, and narrow), and carry various molecular components, thereby forming a meticulous signaling network [27]. EVs from other sources have also been suggested to selectively induce apoptosis in several cancer cells, but not in normal control cells [32,33]. Thus, epididymis-derived EVs not only protect spermatozoa but also defend against the rare occurrence of cancer in epididymis tissue [26,31]. Given the exceptionally low incidence of cancer in epididymal tissue, we hypothesize that epididymal EVs are enriched with anticancer and pro-apoptotic cargo [26]. Here, we propose that epididymal EVs specifically target cancer cells, without affecting normal healthy cells. This proof-of-concept investigation aims at isolating EVs from bull epididymal tissue and assessing their selective anticancer effects on HCC38 cell lines and on MCF-7 cell lines. As a control, non-cancerous neo-human dermal fibroblast (NHDF) cell lines were used [34]. Remarkably, epididymal EVs selectively arrest HCC38 and MCF-7 cells at the G_2_/M phase and induce apoptosis, without affecting NHDF control cells. The cancer-specific activity supports our central hypothesis that epididymal tissue harbors natural anticancer components delivered via EVs and underlines the translational potential of epididymal EVs as a novel, selective, and biologically derived therapeutic strategy for breast cancer.

## 2. Results

### 2.1. Isolation and Characterization of Ep-EVs

**Figure 1:**
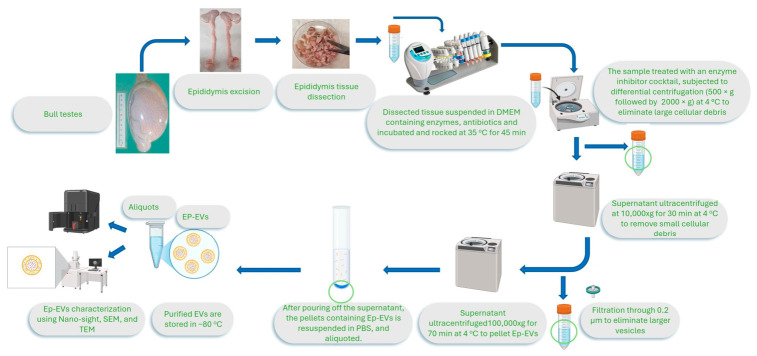
Ep-EV isolation from the epididymis tissues. Epididymis tissues from head (caput—yellow arrow—upper right) to tail (cauda—lower left) were separated and dissected into pieces, and then enzymatically digested using collagenase, and centrifuged to remove all tissue and cellular debris. The supernatant was further ultracentrifuged to pellet Ep-EVs. The Ep-EV pellet was resuspended with PBS, aliquoted and stored at −80 °C until use.

Ep-EVs were isolated from bull testes according to the protocol (Figure 1). Briefly, epididymis tissues were excised, dissected, and exposed to collagenase to obtain a homogenous cellular suspension. After centrifugation to remove cellular debris and larger vesicles, supernatants were ultracentrifuged to obtain EV pellets, which were suspended in PBS and stored at −80 °C until use.

**Figure 2:**
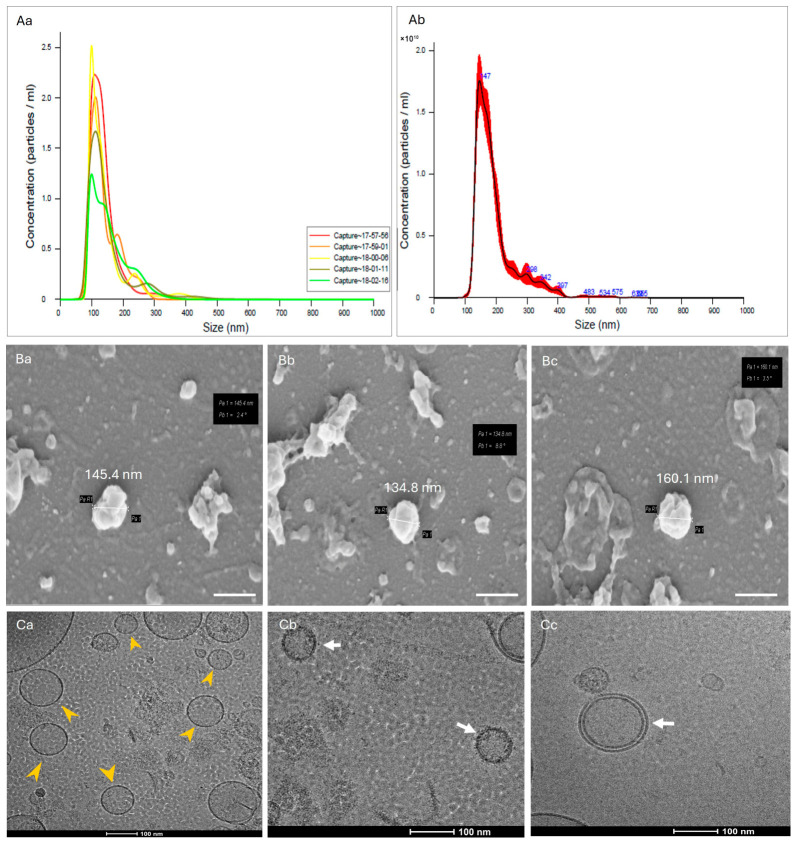
Characterization of Ep-EVs. The size, concentration, and morphology of Ep-EVs isolated from epididymal tissues were determined using NanoSight (Malvern NS300) that uses technology of nanoparticle tracking analysis (NTA) by capturing 5 reads of the Ep-EV suspension (**Aa**) and calculation of the mean of all reads (**Ab**) which was approximately 147nm. Scanning electron microscopy (SEM) analysis of Ep-EVs identified a typical cup-shaped structure (**Ba**–**Bc**) (scale bar = 200 nm), while TEM analysis (**Ca**–**Cc**) showed their spherical-shaped morphology with well-defined edges (yellow arrows; (**Ca**)). The white arrows point to the double-layered membrane of Ep-EVs (**Cb**,**Cc**); scale bar = 100 nm.

The isolated Ep-EVs have a mean size of about 150 nm, characteristic of small EVs, as shown in Figure 2. Ep-EV isolated batches were diluted to 2.5 × 10^11^ Ep-EVs/mL; a stock of Ep-EV suspension was used in all experiments performed in this study. Figure 2 also shows the morphologies of Ep-EVs as visualized by scanning electron microscopy (SEM) and transmission electron microscopy (TEM).

The size distribution was narrow, with most Ep-EVs showing a mean diameter peak of approximately 150 nm. SEM analysis confirmed the classical cup-shaped morphology of EVs, while TEM confirmed the spherical morphology with well-defined edges, with a uniform shape and surface topology consistent with known features of small EVs. Together, these observations provided strong evidence for successful and reproducible isolation of Ep-EVs.

Ep-EVs are typically in the nanoscale size range, and vesicular particles exhibit established rounded lipid bilayer membrane morphologies [18,35,36,37,38]. In the present study, the isolated Ep-EVs were characterized morphologically by electron microscopy, demonstrating a typical lipid bilayer membrane structure and a size distribution ranging between 80 and 150 nm. This size range overlaps with the upper spectrum commonly attributed to small EVs and the lower spectrum of larger extracellular vesicles, such as microvesicles.

### 2.2. Effect of Ep-EVs on Viability and Morphology of Breast Cancer Cells

**Figure 3:**
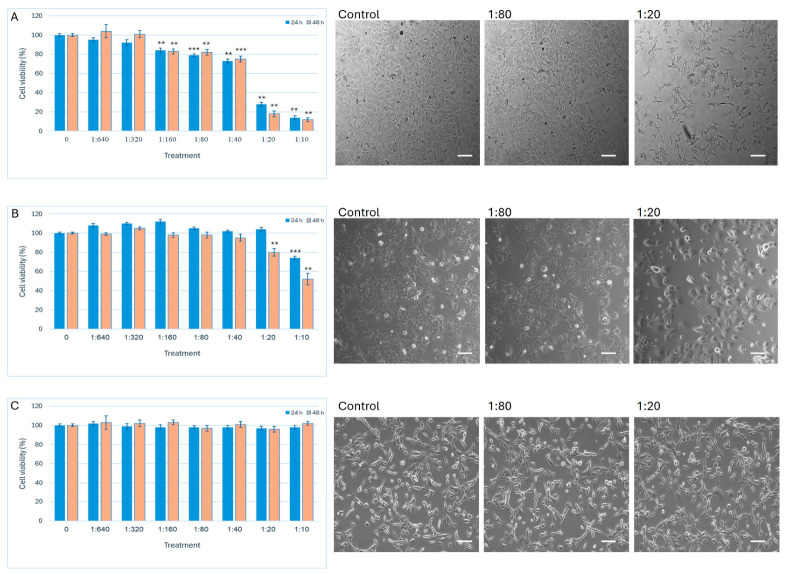
Effect of Ep-EVs on cell viability and morphology. HCC38 (**A**), MCF-7 (**B**), and fibroblast (**C**) cells were treated overnight with Ep-EVs at various dilutions (0 (PBS), 1:640, 1:320, 1:160, 1:80, 1:40, 1:20, 1:10), after which cell viability was determined using the XTT assay. The effect of Ep-EV 1:80 and 1:20 dilutions on cell morphology was assessed after 48 h and compared to control samples treated with PBS only. The results are represented as the mean ± SD of three independent experiments (*n* = 3). Statistical significance across treatment groups was first evaluated using one-way ANOVA, followed by two-tailed unpaired *t*-tests comparing each treatment to the control. Asterisks denote statistical significance relative to the control group: ** *p* < 0.01, and *** *p* < 0.001. Representative light microscopy images demonstrate cell morphology. Scale bar = 20 μm.

The study investigates the effect of Ep-EVs isolated from bull epididymal tissue on breast cancer cells. Two breast cancer cell lines (MCF-7 and HCC38) were utilized in addition to normal fibroblast cells. To evaluate the effect of isolated Ep-EVs on cell viability and morphology, HCC38 and MCF-7 cell lines were treated with different concentrations of Ep-EVs (Figure 3). A significant decrease in cell viability was observed among HCC38 cells treated with ≥1:160 dilutions of Ep-EVs and MCF-7 cells treated with ≥1:20 dilutions of Ep-EVs for 24 h and 48 h. In sharp contrast, high concentrations of Ep-EVs did not affect immortalized fibroblast cells (Figure 3C). A minor increase in cell viability was observed among cells treated with low concentrations of Ep-EVs. HCC38 and MCF-7 cells, which are adherent epithelial cells that grow in a monolayer as single cells or as clusters, detached after exposure to high concentrations of Ep-EVs. Normal fibroblasts, which are elongated, flat, spindle-shaped cells, maintained their morphology even after treatment with high Ep-EV concentrations.

Ep-EV treatment caused a decrease in the viability of HCC38 and MCF-7 cells, as well as triggering shrinkage and detachment of adherent grown cells seen in high Ep-EV treatment compared to the control cells. These results indicate that Ep-EVs significantly reduce cell viability in vitro. To further investigate the underlying mechanism, apoptosis assays were performed on cells.

### 2.3. Apoptotic Effect of Ep-EVs on Breast Cancer Cells

**Figure 4:**
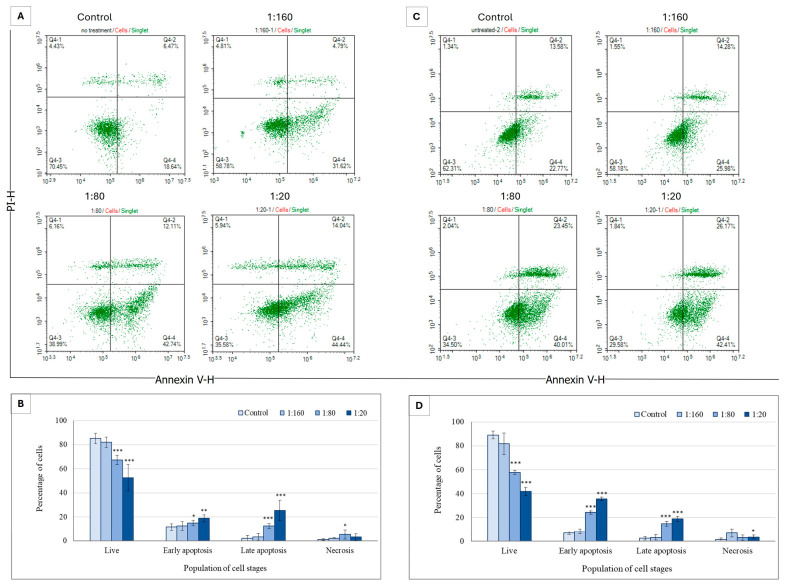
The apoptotic effect of Ep-EVs on HCC38 breast cancer cells. (**A**,**C**) Represent raw data of flow cytometry dot plots of cell apoptosis, and (**B**,**D**) quantification of the proportion of cells in different stages of apoptosis following 24 h (**A**) or 48 h (**C**) treatment of HCC38 cells with Ep-EVs (0 (PBS), 1:160, 1:80, 1:20) and staining with V-annexin-FITC and propidium iodide. The results are presented as the mean ± SD of three independent experiments (*n* = 3). Overall differences between treatment groups were assessed using one-way ANOVA, followed by two-tailed Welch’s *t*-test comparing each treatment condition to the control group. Asterisks denote statistical significance relative to the control group: * *p* < 0.05, ** *p* < 0.01, *** *p* < 0.001.

To determine whether Ep-EVs trigger cancer cell death through a controlled, genetically regulated process (i.e., apoptosis), breast cancer cells were treated with Ep-EVs for 24 h or 48 h and analyzed by flow cytometry following cell staining with annexin V and PI. HCC38 cells exposed to Ep-EVs for 24 h or for 48 h underwent apoptosis, with the number of live cells negatively correlating and the number of early and late apoptotic cells positively correlating with Ep-EV concentrations. In contrast, the number of necrotic cells was low in all Ep-EV-treated samples (Figure 4).

The highest percentage of live HCC38 cells was measured in the control samples, whereas the percentage of live cells decreased and the percentages of early and late apoptotic cells increased in Ep-EVs in a concentration-dependent manner. In contrast, the percentage of necrotic cells remained unchanged with the different Ep-EV treatments, indicating that Ep-EVs induce apoptosis and not necrosis.

**Figure 5:**
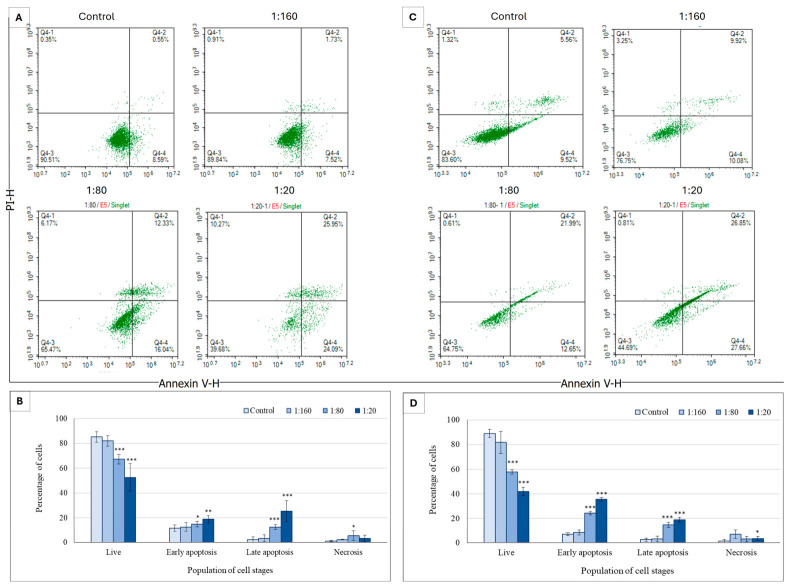
Effect of Ep-EVs on apoptosis of MCF-7 breast cancer cells. (**A**,**C**) Represent raw data of flow cytometry dot plots of apoptotic cells, and (**B**,**D**) quantification of the proportion of cells in different stages of apoptosis following 24 h (**A**) or 48 h (**C**) treatment of MCF-7 cells with Ep-EVs (0 (PBS), 1:160, 1:80, 1:20) and staining with V-annexin-FITC and propidium iodide. The annexin V-FITC and propidium iodide signals were monitored by flow cytometry. The results are presented as the mean ± SD of three independent experiments (*n* = 3). Differences between treatment groups were first evaluated using one-way analysis of variance (ANOVA), followed by two-tailed Welch’s *t*-test comparing each treatment condition with the control group. Asterisks denote statistical significance relative to the control group: * *p* < 0.05, ** *p* < 0.01, *** *p* < 0.001.

To establish this effect in another cell type or breast cancer, cells of the MCF-7 breast cancer cell line were treated with Ep-EVs for 24 h or 48 h and analyzed by flow cytometry following cell staining with annexin V and PI (Figure 5).

Similar effects were observed in MCF-7 cells; 48 h treatment with Ep-EVs triggered an apoptotic effect similar to that of the 24 h treatment, although to a lesser degree, with the highest percentage of early apoptotic cells measured in samples treated with 1:20 Ep-EVs for 48 h, and the highest percentage of late apoptotic cells measured in the samples treated with 1:20 Ep-EVs for 24 h (Figure 5). The percentage of live cells decreased as the Ep-EV concentration increased, as seen in the percentages of early and late apoptotic cells with 24 h treatment. A significant increase in the percentage of late apoptotic cells was seen in samples treated with 1:20 Ep-EVs compared to the control.

**Figure 6:**
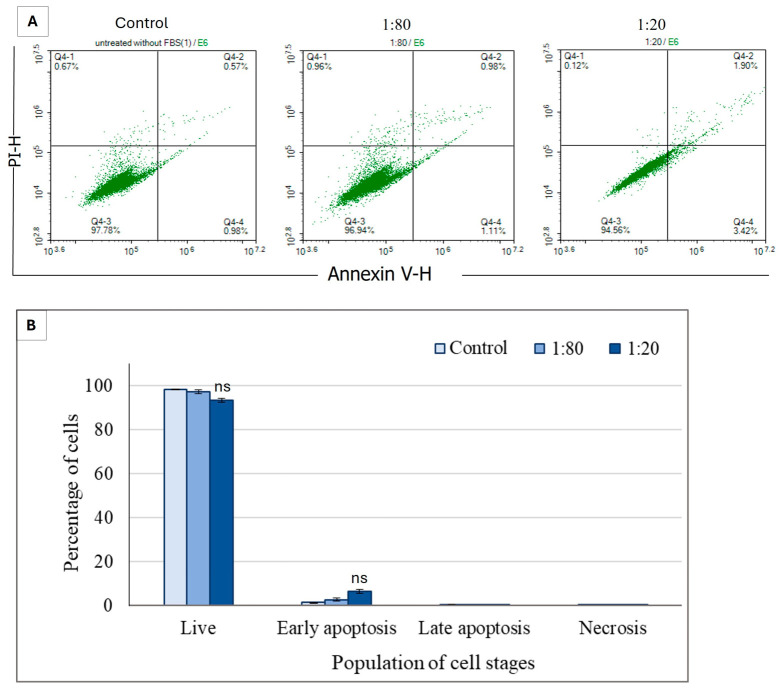
Effect of Ep-EVs on apoptosis of normal fibroblast cells. (**A**) Represents raw data of flow cytometry dot plots of cell apoptosis, and (**B**) quantification of the proportion of cells in different stages of apoptosis following 48 h treatment of normal fibroblast cells with Ep-EVs (0 (PBS), 1:80, 1:20) and staining with V-annexin-FITC and propidium iodide. The results are presented as the mean ± SD of three independent experiments (*n* = 3). Differences between treatment groups were evaluated using one-way analysis of variance (ANOVA), followed by two-tailed Welch’s *t*-test comparing each treatment condition with the control group. “ns” indicates no statistically significant difference relative to the control group (*p* ≥ 0.05).

In the NHDF cultures, more than 95% of the cells remained viable across the tested Ep-EV concentrations, with only a slight concentration-dependent increase in the percentage of early apoptotic cells observed following 48 h exposure to Ep-EVs. Overall, Ep-EV treatment at both 1:80 and 1:20 dilutions had no significant apoptotic effect on fibroblasts compared to the control cells treated with PBS only (Figure 6).

### 2.4. Effect of Ep-EVs on the Cell Cycle of Breast Cancer Cells

**Figure 7:**
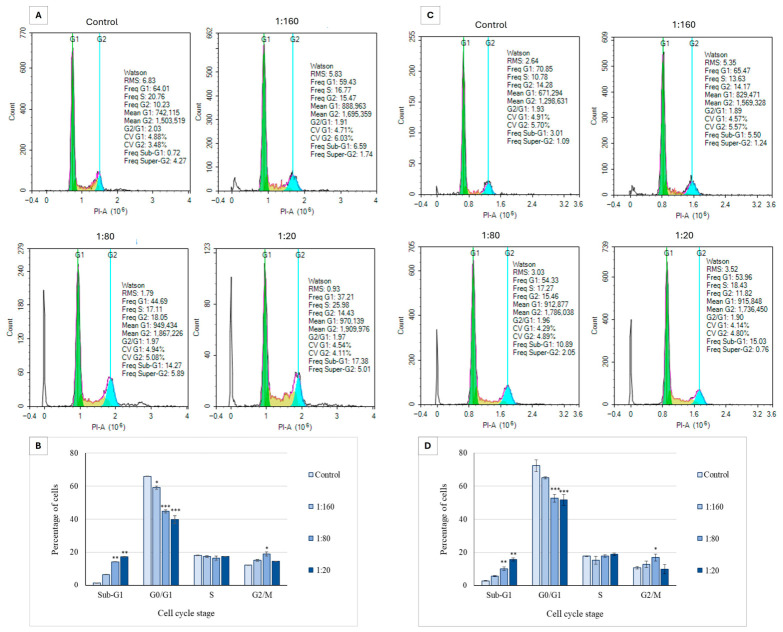
The effect of Ep-EVs on cell cycle distribution in breast cancer cell lines HCC38 (**A**,**B**) and MCF-7 (**C**,**D**). Cells in culture were treated with various Ep-EV dilutions (0 (PBS), 1:160, 1:80, 1:20) for 48 h and then fixed and treated with propidium iodide, then collected and analyzed by flow cytometry for cell cycle analysis. Histograms represent raw data of cell cycle distribution by flow cytometry analysis (**A**,**C**). Quantitative analysis of cell distribution in the cell cycle phases was calculated and presented in (**B**,**D**). The results are represented in the form of mean ± SD from three independent experiments (*n* = 3). Differences among treatment groups were first evaluated using one-way analysis of variance (ANOVA), followed by two-tailed Welch’s *t*-test comparing each treatment condition with the control group. Asterisks denote statistical significance relative to the control group: * *p* < 0.05, ** *p* < 0.01, *** *p* < 0.001.

**Figure 8:**
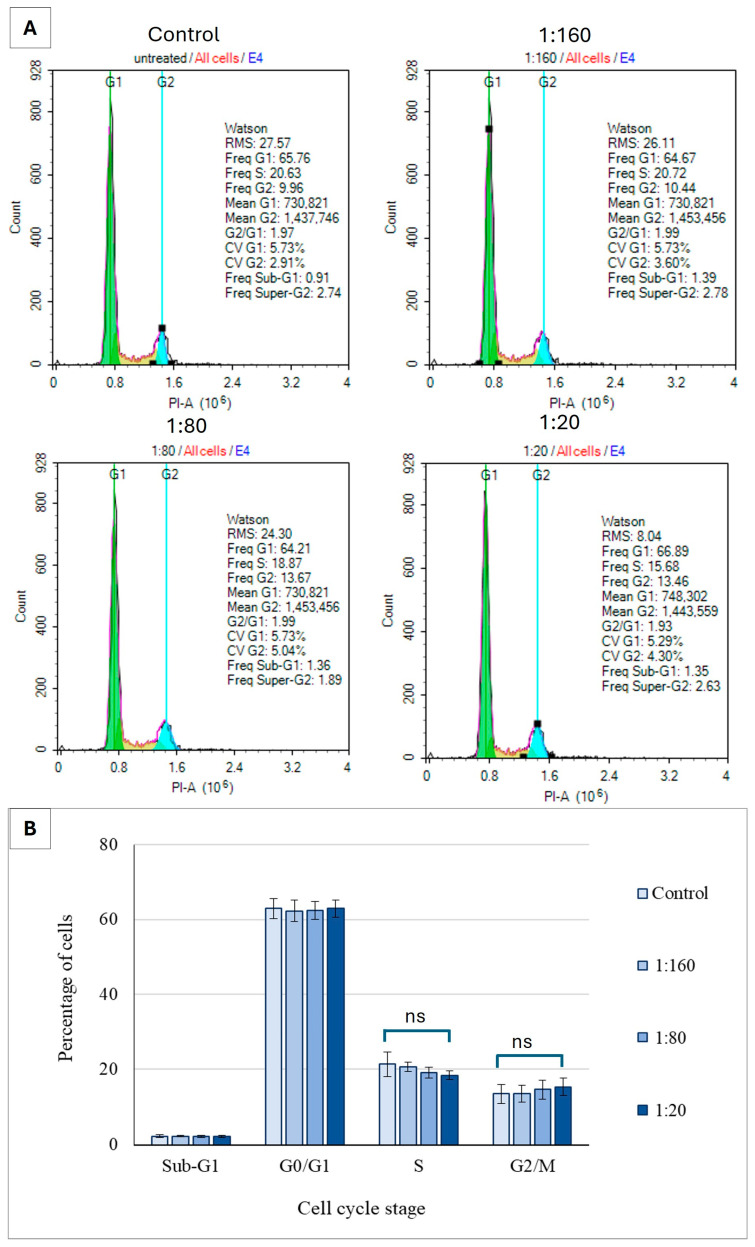
The effect of Ep-EVs on cell cycle distribution of normal fibroblast cells. Cultured fibroblast cells were treated with various dilutions of Ep-EVs (0 (PBS), 1:160, 1:80, 1:20) for 48 h and then fixed and treated with propidium iodide and analyzed by flow cytometry. (**A**) Histograms represent raw data of cell cycle distribution. (**B**) Quantitative analysis of cell distribution across the cell cycle phases. The results are presented as the mean ± SD of three independent experiments (*n* = 3). Differences between treatment groups were evaluated using one-way analysis of variance (ANOVA), followed by two-tailed Welch’s *t*-test comparing each treatment condition with the control group. “ns” indicates no statistically significant difference relative to the control (*p* ≥ 0.05).

Our findings indicate that Ep-EVs induce apoptosis in breast cancer cells (HCC38 and MCF-7), demonstrating that their cytotoxic activity operates via programmed cell death rather than necrotic pathways. Cell cycle analysis was employed to determine how Ep-EVs suppress cell proliferation or affect their growth regulation. As shown in Figure 7, Ep-EV treatment activated the apoptotic cascade in both HCC38 and MCF-7 cell lines, as demonstrated in the sub-G1 phase. While there was no significant effect on the arrested cells observed at the S phase among the different Ep-EV treatments, a concentration-dependent increase in the percentage of cells arrested in the G_2_/M phase was observed following treatment with 1:160 and 1:80 Ep-EV concentrations in both HCC38 and MCF-7 cell lines. The percentage of cells following treatment with 1:20 Ep-EVs was lower compared to the other treatments (Figure 7B,D). In sharp contrast, Ep-EV treatments at all tested concentrations did not induce any significant changes in the cell cycle distribution of normal fibroblast cells (Figure 8A,B).

Collectively, the data indicate that Ep-EVs promote apoptosis, as evidenced by the accumulation of DNA-fragmented cells, with this effect becoming more pronounced at higher Ep-EV concentrations. The higher the percentage of cells seen in the sub-G_1_ phase (non-living cells), the more apoptosis is occurring. Also, this experiment demonstrated that Ep-EVs induced G_2_/M cell cycle arrest at low concentrations (1:160 and 1:80). Interestingly, cells are not arrested very much when treated with Ep-EVs at higher concentrations (1:20) (Figure 7B,D). These results indicate that Ep-EVs can induce cell cycle arrest in the G_2_/M phase and apoptosis, leading to the inhibition of HCC38 and MCF-7 cell proliferation.

To assess whether Ep-EVs influence cell cycle progression in normal cells, the distribution of fibroblast cell populations across the G_0_/G_1_, S, and G_2_/M phases was determined at various Ep-EV treatments (Figure 8).

As seen, Ep-EV treatment at the tested concentrations did not cause a significant alteration in the distribution of cells across the cell cycle phases (G_0_/G_1_, S, and G_2_/M), indicating that the Ep-EVs do not affect cell cycle progression under these conditions. To verify the change in G_2_/M phase at the molecular level as cells were treated with Ep-EVs, the levels of key regulatory proteins may be examined in normal and cancer cells by Western blotting.

### 2.5. The Effect of Ep-EVs on the Expression of p53

**Figure 9:**
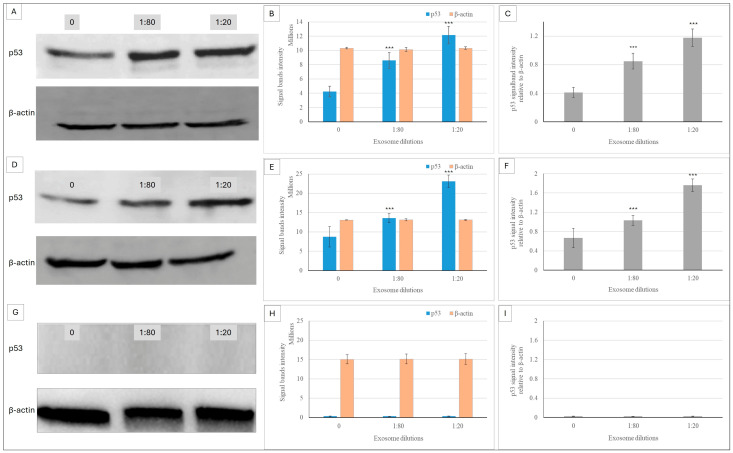
Ep-EVs modulate p53 protein expression in breast cancer cells and normal fibroblasts. Cells of HCC38 (**A**–**C**), MCF-7 (**D**–**F**), and normal fibroblast cell lines (**G**–**I**) were treated with Ep-EVs for 48 h, their lysates were separated on SDS-PAGE, and proteins were transferred to nitrocellulose membranes, which were then reacted with anti-p53 antibody and anti-HRP secondary antibody. (**A**,**D**,**G**) present representative Western blots: untreated cells (0), cells treated with Ep-EVs diluted 1:80, and cells treated with Ep-EVs diluted 1:20. β-actin (43 kDa) served as the internal housekeeping protein control. (**B**,**E**,**H**) present intensity quantification of the protein bands, and (**C**,**F**,**I**) present calculations of p53 relative to β-actin protein band intensities. The results are presented as the mean ± SD of three independent experiments (*n* = 3). Differences between treatment groups were evaluated using one-way analysis of variance (ANOVA), followed by two-tailed unpaired *t*-tests comparing each treatment condition with the control group. Asterisks denote statistical significance relative to the control group: *** *p* < 0.001.

p53 is a central pro-apoptotic regulatory protein, which is a key mechanism by which it acts as a cancer suppressor. It is involved in protecting cells from genetic events, either by gene repair or by mediating cell death. Its expression levels in Ep-EV-treated cancer cell lines were investigated and quantified by Western blotting analysis. An increase in p53 expression was measured following Ep-EV treatment of both breast cancer cell lines, compared to the untreated control (Figure 9). The highest level of p53 expression was measured in cells treated with 1:20 Ep-EVs. In normal fibroblasts, Ep-EVs demonstrated no effect on p53 expression (Figure 9G). Of note, Western blot analysis revealed that p53 expression in normal fibroblasts was below the detection threshold under our conditions, while HCC38 and MCF-7 cells exhibited high basal p53 expression levels as well as higher p53 expression when treated with Ep-EVs.

### 2.6. Ep-EV Uptake by HCC38 and MCF-7 Cells

**Figure 10:**
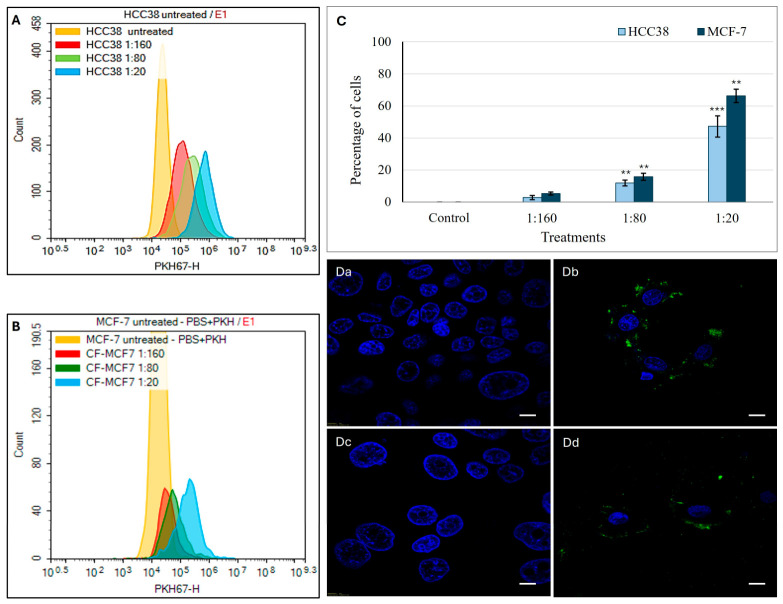
Uptake of PKH67-labeled Ep-EVs by breast cancer cells. HCC38 and MCF-7 cells (1 × 10^5^ cells) were seeded onto chamber slides and cultured overnight, followed by treatment with PKH67-labeled Ep-EVs (2.5 × 10^10^ particles/mL stock concentration) at dilutions of 1:160, 1:80, and 1:20 for 18 h. Representative flow cytometry histograms demonstrating concentration-dependent cellular uptake of PKH67-labeled Ep-EVs in HCC38 (**A**) and MCF-7 (**B**) cells. (**C**) Quantification of Ep-EV uptake expressed as the percentage of PKH67-positive cells in HCC38 and MCF-7 cultures. Data are presented as mean ± SD of three independent experiments (*n* = 3). Statistical analysis was performed using one-way analysis of variance (ANOVA) followed by two-tailed unpaired *t*-tests comparing each treatment group with the respective control. ** *p* < 0.01, and *** *p* < 0.001. (**Da**–**Dd**) Representative confocal microscopy images confirming Ep-EV internalization. Nuclei were counterstained with Hoechst (blue), and internalized PKH67-labeled Ep-EVs are shown in green. (**Da**,**Dc**) Control cells showing a negligible fluorescence signal. (**Db**,**Dd**) Cells treated with Ep-EVs (1:20 dilution) exhibiting cytoplasmic punctate fluorescence consistent with Ep-EV uptake. Scale bar = 10 μm.

To evaluate the internalization efficiency of Ep-EVs by human breast cancer cells, Ep-EVs fluorescently labeled with the lipophilic dye PKH67 were incubated with HCC38 and MCF-7 cells for 18 h. Cellular uptake was quantified by flow cytometry at three serial dilutions (1:160, 1:80, and 1:20) of a 2.5 × 10^11^ particles/mL stock suspension. Flow cytometric analysis demonstrated a clear concentration-dependent increase in Ep-EV uptake in both cell lines (Figure 10). At the lowest concentration (1:160), uptake was minimal, with approximately 2–3% positive HCC38 cells and 4–5% positive MCF-7 cells. Following treatment with 1:80 Ep-EVs, approximately 10–12% of HCC38 and 15–17% of MCF-7 cells were PKH67-positive. At the highest tested concentration of Ep-EVs (1:20), 47–50% of HCC38 cells and 65–67% of MCF-7 cells showed vesicle uptake. Notably, Ep-EV internalization was consistently higher in MCF-7 cells compared to HCC38 cells across all tested concentrations, indicating differential uptake efficiency between the two breast cancer subtypes. Confocal fluorescence microscopy further confirmed the internalization of PKH67-labeled Ep-EVs (Figure 10(Da–Dd)). Control cells displayed negligible green fluorescence, whereas cells treated with Ep-EVs (1:20 dilution) exhibited distinct punctate green fluorescence within the cytoplasm surrounding Hoechst-stained nuclei, consistent with intracellular vesicle uptake. These observations corroborate the flow cytometry data and demonstrate efficient cellular internalization of Ep-EVs.

These findings confirm that bull-derived Ep-EVs are efficiently internalized by human breast cancer cells, supporting their cross-species compatibility and functional internalization.

## 3. Discussion

In this study, small EVs were isolated from bull epididymal tissue. Notably, the Ep-EVs exhibited a selective anticancer effect. The Ep-EVs presented anticancer effects on HCC38 cell lines (human epithelial breast cancer cells) that carry homozygous mutant p53 [39]. Likewise, the Ep-EVs showed anticancer effects on MCF-7 cell lines (human breast cancer cells) that carry homozygous WT p53 [40]. In contrast, the Ep-EVs did not show any effect on control non-cancerous NHDF cell lines [34]. Remarkably, the Ep-EVs selectively inhibit the growth and viability of human breast cancer cells in a time- and concentration-dependent manner, while showing no effect on normal fibroblast cells. These findings support our hypothesis that small EVs derived from tissues with rare cancer incidence carry pro-apoptotic cargo and regulators of survival pathways, enabling them to selectively trigger apoptosis in cancer cells while leaving normal cells unharmed.

SEM and TEM images (Figure 2(Ba–Bc,Ca–Cc)) demonstrated that the fraction of EVs isolated from the epididymis tissue was primarily small EVs [41,42]. Following EV treatments, HCC38 and MCF-7 cells shrank in size (Figure 3A,B), and their proliferation rate and confluence decreased significantly compared to the control. The cells appeared stressed and partially damaged, with severity demonstrating a direct correlation with Ep-EV concentrations. In contrast, no changes in fibroblast proliferation, confluence, or morphology were noted (Figure 3C), suggesting that the effect of Ep-EVs is specific to cancer cells. While the viability of both HCC38 and MCF-7 cells decreased following treatment with a high concentration (1:10) of Ep-EVs, HCC38 cells appeared more sensitive to Ep-EVs (Figure 3A,B), while normal fibroblasts were not affected by Ep-EVs (Figure 3C). Taken together, the results demonstrate that Ep-EVs selectively inhibit breast cancer cell viability while sparing normal fibroblasts. This observation aligns with accumulating evidence that exosomes, a specific subpopulation of small EVs derived from non-tumor sources, can exert significant anticancer activity. Zhao et al. [43] reported that immune-cell-derived exosomes suppress tumor growth through mechanisms such as the activation of cytotoxic T cells and NK cells, delivery of perforin and granzymes, FasL/TRAIL-mediated apoptosis, and modulation of inflammatory signaling, with favorable safety profiles in vivo.

Importantly, whereas these studies largely attribute antitumor effects to immune-mediated cytotoxicity, our findings extend this concept by showing that Ep-EVs—originating from an immune-privileged tissue with a low incidence of malignancy—directly induce G_2_/M arrest and apoptosis in breast cancer cells without affecting normal fibroblasts. These data suggest that specialized tissues may release EVs enriched with intrinsic anticancer cargo, positioning Ep-EVs as a biologically inspired and potentially safe therapeutic platform.

The apoptotic effect of Ep-EVs on HCC38 and MCF-7 cells was also investigated using an annexin V-FITC/PI staining assay [44] (Figure 4 and Figure 5). HCC38 (Figure 4) and MCF-7 (Figure 5) cell exposure to Ep-EVs resulted in a clear, concentration- and time-dependent induction of apoptosis, with minimal necrotic activity. This demonstrates that the cytotoxic effect is primarily mediated through a programmed cell death mechanism. Of note, the percentage of untreated HCC38 control cells in early apoptosis was relatively high (Figure 4D), and may have been the result of stress starvation due to serum-free media. MCF-7 cells exhibited a similar but slower and less extensive response to Ep-EVs, characterized by a moderate rise in early apoptosis and limited progression to late apoptotic stages, even after 48 h of treatment. The differences in sensitivity to Ep-EV treatment between the two cell lines may reflect their overall mutational landscape and corresponding molecular subtype-specific signaling networks. HCC38, a triple-negative line lacking classical ER/PR/HER2 signaling, may have fewer hormone receptor-mediated pro-survival inputs, potentially increasing susceptibility to pro-apoptotic stimuli. By contrast, ER-positive MCF-7 cells engage estrogen-driven PI3K/Akt survival pathways that can partially buffer apoptotic signals [45,46], possibly requiring higher Ep-EV concentrations to drive more cells into the early and late stages of apoptosis. Most importantly, no significant apoptotic or necrotic effects were observed in normal fibroblast cells exposed to the same concentrations of Ep-EVs (Figure 6). Taken together, Ep-EVs exert selective cytotoxicity toward cancer cells while exhibiting high compatibility with normal cells. Several studies assessing the effects of exosomes derived from non-tumor sources, including MSCs [47], natural killer cells [24] and plant cells [48,49], also demonstrated their anticancer effects on cancer cells in vitro. However, similar to Ep-EVs, natural killer exosomes [50] and some plant exosomes have a selective anticancer effect while not affecting normal cells [48,49]. This selective activity may possibly be due to enhanced EV uptake or to the higher sensitivity of cancer cells associated with their distinct membrane structure [44,51].

The effect of Ep-EVs on the cell cycle phases of breast cancer cells was also significant. In both HCC38 and MCF-7 cells (Figure 7B,D), treatment with increasing Ep-EV concentrations resulted in marked alterations in cell cycle progression, accompanied by the appearance of a sub-G1 population, indicative of apoptotic DNA fragmentation. The magnitude of these changes was slightly greater in HCC38 than in MCF-7 cells, consistent with the differential apoptotic sensitivity described above. HCC38 cells appeared more vulnerable to Ep-EV-induced stress, with slightly stronger G_2_/M arrest, alongside stronger effects on apoptosis and on cell viability. Cell cycle arrest occurring during cell division is suggestive of cellular damage or replication errors that are beyond repair. Arrest in the G_2_/M phase, in particular, indicates that the DNA damage checkpoint has been activated and that the extent of genomic damage is irreparable, preventing the cell from entering mitosis [52,53]. An increase in the sub-G1 population, indicative of DNA fragmentation, has been observed upon exposure to various anticancer agents and correlates with inhibition of cell cycle progression and induction of apoptosis [54,55]. Interestingly, no significant change was observed in the cell cycle phases of normal fibroblasts at any of the tested Ep-EV concentrations (Figure 8), further highlighting the selective Ep-EV-driven modulation of cell cycle checkpoints in cancer cells by upregulating pro-apoptotic proteins and downregulating anti-apoptotic factors. Apoptosis in normal cells is tightly regulated, whereas in cancer cells it is often disrupted, rendering cancer cells more vulnerable to certain selective anticancer agents [56].

The apoptotic effect of Ep-EVs is likely mediated via p53—often termed the ‘guardian of the genome’—which, in response to cellular stress or DNA damage, governs cell cycle arrest and the execution of apoptosis [57]. Indeed, in both cell lines, Ep-EV treatment resulted in a concentration-dependent increase in p53 protein expression, while no p53 expression was detected in normal fibroblast cells, even following Ep-EV treatment. Generally, under physiological conditions, p53 protein levels are tightly regulated and maintained at very low concentrations in normal cells, as demonstrated in fibroblasts [58]. In contrast, basal p53 levels are frequently higher in cancer cells, as seen in HCC38 and MCF-7 cells (Figure 9). In HCC38 cells, p53 carries a mutation that results in the accumulation of a nonfunctional, yet stable protein that loses its tumor-suppressive capacity [59,60]. Although nonfunctional, p53 protein levels increased upon Ep-EVs exposure, likely due to its stabilization through phosphorylation events that prevent degradation [61]. MCF-7 cells, which express wild-type p53, also exhibited elevated basal levels of p53 protein compared to normal fibroblasts. HCC38 cells, despite harboring a mutant p53, exhibited robust apoptotic responses following Ep-EV treatment, accompanied by an apparent increase in p53 protein levels. In MCF-7 cells, Ep-EVs activated the p53 axis, whereas in HCC38 cells, they induced p53-independent apoptotic signaling. Taken together, these findings indicate that Ep-EVs can trigger apoptosis in breast cancer cells regardless of whether p53 is wild-type or mutant. The ability of Ep-EVs to induce apoptosis irrespective of p53 status highlights the therapeutic potential of Ep-EVs as a versatile anticancer agent, owing to their diverse cargo components targeting multiple mechanisms. While the precise cargo responsible for these effects remains to be defined, the mechanistic framework proposed here provides a biologically plausible basis for the observed selective anticancer activity. Future investigations will incorporate comprehensive molecular characterization of Ep-EVs, including established positive and negative EV markers [23,62].

Consistent with the aforementioned mechanistic interpretation, the following uptake findings provide functional support at the cellular entry level. The functional assays may be related to the extent of Ep-EV internalization. Flow cytometry analyses and fluorescence microscopy revealed pronounced internalization of PKH67-labeled Ep-EVs by HCC38 and MCF-7 cells, with a strong intracellular fluorescent signal distributed throughout the cytoplasm and the perinuclear space (Figure 10). These findings were supported by flow cytometry uptake quantification, where HCC38 and MCF-7 cells had a high level of uptake (Figure 10). This internalization dynamic may explain the responses observed in HCC38 and MCF-7 cells and suggests similar endocytic machinery in the two cell lines. EVs are known to interact with recipient cells via a number of endocytosis pathways, including clathrin-mediated endocytosis, caveolin-mediated endocytosis, micropinocytosis and direct membrane fusion [63]. Collectively, these findings demonstrate efficient and concentration-dependent uptake of bull Ep-EVs by human breast cancer cells, supporting cross-species compatibility and functional internalization. Nevertheless, the precise molecular mechanisms governing Ep-EV uptake by mammalian cells remain unclear, largely due to the limited characterization of EV cargo and the membrane receptors or surface proteins that mediate their cellular entry.

Certain limitations of this study should be acknowledged. While the findings point to a selective effect of Ep-EVs on cancer cells, the evidence is still preliminary, as validation across a broader range of cancer and normal cell lines will still be needed. Additional studies are therefore required to determine whether this selectivity represents a general cancer-specific phenomenon. Additionally, future work employing breast cancer xenograft models in nude mice will be essential to assess the in vivo antitumor effect of Ep-EVs.

Another important limitation is the cross-species nature of the experimental model, in which bovine-derived EVs are applied to human cancer cells, potentially involving species-specific differences in molecular pathways. For example, the p53 protein sequence is not identical between *Bos taurus* and *Homo sapiens*, and species-specific variations may limit the generalizability of our findings. However, according to multiple sequence alignment, p53 is highly conserved across species [29], with ≥60% sequence identity and strong structural similarity, particularly within its DNA-binding and functional domains. This conservation supports its role in fundamental cellular processes such as DNA repair, apoptosis, and cell cycle regulation.

In addition, EVs in general are known to mediate intercellular communication in a largely species-independent manner. Consistent with this, bovine Ep-EVs were efficiently internalized by human breast cancer cells in our study, supporting functional cross-species compatibility. Therefore, we propose that Ep-EVs may deliver conserved regulatory cargo (e.g., proteins and miRNAs) capable of modulating p53-dependent pathways in recipient human cancer cells, leading to apoptosis and cell cycle arrest. Nevertheless, species-specific differences may still influence the magnitude or specificity of these effects, and further studies using human-derived EVs will be required to validate their translational relevance. In conclusion, Ep-EVs effectively inhibit the proliferation of HCC38 and MCF-7 breast cancer cells, while upregulating p53, resulting in G_2_/M cell cycle arrest and apoptosis induction. The effects of Ep-EVs on breast cancer cells highlight their potential as a natural, biocompatible and selective anticancer agent, capable of discriminating between breast cancer cells and normal fibroblasts. These results establish a strong experimental foundation for consideration of Ep-EVs as a therapeutic approach for breast cancer disease, which may subsequently constitute a platform for the treatment of other types of cancer. Further studies incorporating in vivo validation, biodistribution profiling, and immune response assessment will be necessary to evaluate their translational and therapeutic relevance. Notably, this study provides the first proof-of-concept that Ep-EVs exhibit intrinsic anticancer activity, which may contribute to the remarkably low incidence of cancer in epididymal tissues.

## 4. Materials and Methods

In line with the Minimal Information for Studies of Extracellular Vesicles (MISEV) guidelines issued by the International Society for Extracellular Vesicles (ISEV) [64], we used the collective and operational term “extracellular vesicles (EVs)” to describe the isolated nano-sized particles throughout this study.

### 4.1. Epididymis Separation

Epididymal tissue was separated from the testis and dissected into 2 mm pieces in 10 mm plates, using a scalpel and/or curved scissors. The procedure was performed aseptically in a biological safety cabinet. The pieces were then washed with cold Dulbecco’s modified Eagle’s medium (DMEM) (Cat. No. No. 01-106-1A; Biological Industries, Beit Haemek, Israel) supplemented with glucose (1000 mg/L), penicillin and streptomycin (1% final concentration, IMBH, Beit Haemek, Israel). Once the dissected epididymis tissue pieces became clear of cloudy sperm fluid, the tissue pieces were suspended in a final volume of 3 mL DMEM 2X dissociation medium containing 80 μL 25X collagenase (Cat. C6885-, Sigma Aldrich, Rehovot, Israel), and 40 μL 50X DNase I (Cat. 10104159001, Sigma Aldrich, Rehovot, Israel). Samples were then rocked (30 rpm) on a shaker at 35 °C, for 30–45 min, and passed through a 10 mL plastic pipette until a homogenous suspension was obtained. Collagenase activity was then terminated by the addition of a protease and phosphatase inhibitor cocktail (1%) (Cat. PPP 2020, Sigma Aldrich, Rehovot, Israel).

### 4.2. Ep-EV Isolation

The dissociated tissue suspension was centrifuged at 500× *g* and 4 °C, for 5 min, to pellet the cells and the remaining fibers or cohesive tissue fragments. Thereafter, the supernatant was transferred to a clean 50 mL tube and centrifuged at 2000× *g* and 4 °C, for 10 min, to pellet and discard the large cellular debris. Then, the supernatant was ultracentrifuged for 30 min, at 10,000× *g* and 4 °C, to further remove small cellular debris. Subsequently, the supernatant was passed through a 0.2 μm filter to eliminate larger vesicles. Cold PBS was added to the filtrate to bring the volume to 32 mL. The suspension was ultracentrifuged for 70 min, at 100,000× *g* and 4 °C to pellet the Ep-EVs [65]. After pouring off the supernatant, the pellets were resuspended in PBS (200 µL to resuspend the pellet in a tube and another 200 µL to wash pellet residua off the tube walls) and pooled in a single tube. The volume of the pooled Ep-EV pellets was then brought to approximately 38 mL with cold PBS, and samples were ultracentrifuged to eliminate contaminating proteins. The pellet was resuspended in 1500 μL PBS, with 750 μL PBS first used to resuspend the pellet, and then another 750 μL PBS used to wash and collect the remaining Ep-EVs in the pellet tube. Samples were divided into aliquots of 200 µL and stored at −80 °C until use.

### 4.3. Nanoparticle Tracking Analysis

Isolated Ep-EVs were thawed and diluted with filtered PBS 1:1000 for nanoparticle tracking analysis. Ep-EV concentration and size were measured using the NanoSight LM14C system (Malvern Panalytical Ltd., Marvern, UK), according to the manufacturer’s guidelines. Signals were recorded for 30 s [66], and then analyzed with the NanoSight software NTA VER 3.4 using the Stokes–Einstein equation. All Ep-EV isolate batches were diluted to stock suspensions of 2.5 × 10^11^ particles/mL, which were then used in all experiments performed in this study.

### 4.4. Preparation of Ep-EVs for Scanning Electron Microscopy (SEM)

Isolated Ep-EVs (5 μL of suspension) were applied onto membrane filter disks and incubated in 3% glutaraldehyde (prepared in phosphate buffer, PO_4_) for 1 h at room temperature. The fixative was then aspirated, and the samples were washed four times with phosphate buffer at 10 min intervals. Dehydration was performed by gradually replacing the buffer with increasing concentrations of ethanol (10%, 30%, 50%, 70%, and 90%) to avoid osmotic shock. The final dehydration step was carried out in 100% ethanol for 1 h with three changes. Samples were then dried using liquid CO_2_, sputter-coated with gold, and imaged using a Zeiss Supra 55VP (Carl Zeiss AG, Oberkochen, Germany) scanning electron microscope.

### 4.5. Preparation of Ep-EVs for Transmission Electron Microscopy (TEM)

Isolated Ep-EVs (5 μL of suspension) were deposited onto carbon-coated copper grids (Formvar/carbon support) and allowed to adsorb for 20 min at room temperature. Excess liquid was removed using filter paper. Grids were then fixed in a solution containing 2.5% glutaraldehyde, 4% paraformaldehyde (PFA), and 0.25% tannic acid in PBS for 10 min. After fixation, the grids were washed with distilled water and allowed to dry. The samples were examined using Thermo-Fisher Talos F200C, FEG-equipped high resolution-TEM at 73000x. 

### 4.6. Cell Culture

HCC38 and MCF-7 human breast cancer cell lines and NHDFs (American Type Culture Collection, (ATCC) CRL-2314, HTB-22, PCS-201-010, respectively) were thawed and cultured in T75 flasks, at 37 °C, with 5% CO_2_. HCC38 cells were cultured in RPMI-1640 medium (Cat. No. 01-152-1A; Sartorius, Beit Haemek, Israel) containing 2 mM L-glutamine, 10 mM HEPES, 1 mM sodium pyruvate, 4500 mg/L glucose and 1500 mg/L sodium bicarbonate. MCF-7 and NHDF cells were cultured in Dulbecco’s modified Eagle’s medium (DMEM) (Cat. No. 01-106-1A; Sartorius, Beit Haemek, Israel) containing 4 mM L-glutamine, 4500 mg/L glucose, 1 mM sodium pyruvate and 1500 mg/L sodium bicarbonate. Both media were supplemented with fetal bovine serum (Cat. No. BWS181H500; IMBH, Beit Haemek, Israel; 10% final concentration), penicillin and streptomycin (IMBH, Beit Haemek, Israel, 1% final concentration).

### 4.7. Cell Viability

The mitochondrial metabolism of cells, which reflects their viability, was measured using the XTT assay. Cells (5 × 10^3^ cells/well) were seeded in 96-well plates and incubated for 24 h, in 100 µL DMEM supplemented with 10% fetal bovine serum. After aspirating the medium, the adhered cells were washed with PBS and then incubated (37 °C, 2 h) with 50 µL serum-free medium and Ep-EVs, and then suspended in PBS at dilutions of 0 (PBS only), 1:640, 1:320, 1:160, 1:80, 1:40, 1:20, and 1:10. Then, another 50 μL of serum-free medium was added to each well. Cell viability was assessed using the XTT kit (Cat. No. 20-300-1000; Sartorius, Biological Industries, Ltd., Beit Haemek, Israel), as per the manufacturer’s protocol. Signals were spectrophotometrically measured at 492 nm (reference wavelength 630–690 nm) by a Varioskan LUX Multimode Microplate Reader (Thermo Scientific, Singapore). Each test was performed in triplicate, and three independent tests were conducted. Viability was calculated using the background-corrected absorbance, calculated as follows: Cell viability (%)  =  [absorbance of test samples]/[absorbance of control] × 100.

### 4.8. Apoptosis Assay

Apoptotic and dead cells were quantified using an Annexin V FITC Detection kit (Cat. No. MBL-4700; Mebcyto^®^ Apoptosis kit, MBL International, Woburn, MA, USA)), according to the manufacturer’s recommended procedure, and flow cytometry. HCC38 and MCF-7 cells (2 × 10^5^) were seeded in complete medium in 6-well plates. After 24 h, the medium was discarded, the cells were washed with PBS and then incubated at 37 °C for 2 h with 750 μL serum-free medium and Ep-EVs (dilutions of 0 (treated with PBS), 1:160, 1:80, and 1:20). Thereafter, another 700 μL serum-free medium was added, and cells were incubated for 24 h at 37 °C. After treatment with 500 μL trypsin for 2 min, and neutralization with complete medium, both floating and adhered cells were collected and centrifuged at 1200 rpm. Pellets were then washed once with PBS and suspended in 85 μL binding buffer (Cat. No. 00-0055-43; Rhenium, Modiin, Israel). Subsequently, 10 μL annexin V-FITC and 5 μL propidium iodide (PI) were added, and samples were incubated in the dark for 15 min at room temperature. The signal generated by annexin V-FITC, a marker of apoptosis, was detected in the FITC signal detector FL1, while the signal generated by PI, a marker of dead cells, was monitored by the detector for phycoerythrin emissions (FL2 or FL3). Results were analyzed using the Novocyte flow-cytometer, Novoexpress program v1.5.0. (Lumitron Ltd., Petah Tikva, Israel), which provides a variety of plots and gates for flow cytometry data analysis, including elliptical gates and dot and density plots.

### 4.9. Cell Cycle Analysis

HCC38 and MCF-7 cells were seeded in complete medium in 6-well plates, at a density of 2 × 10^5^ cells/well, and incubated overnight, at 37 °C. Cells were then washed with PBS and incubated for 2 h in 750 μL serum-free medium and Ep-EVs, (dilutions of 0 (PBS only), 1:160, 1:80, 1:20) in triplicate. Thereafter, 700 μL serum-free medium was added, and cells were further incubated for 48 h. The cells were then trypsinized, washed with ice-cold PBS and centrifuged. Pellets were suspended and vortexed in 2.5 mL 70% EtOH. The cell mixture was then placed on ice for 15 min and centrifuged at 1500 rpm for 5 min. Pellets were suspended in 500 μL PI solution (1 mL Triton 1% (Cat. No. X100-T8787; Merck, Sigma Aldrich, Rehovot, Israel), 1 mL PI stock (Cat. No. P4864; Merck, Sigma Aldrich, Israel), 2 mL RNase (Cat. No. 1010942001; Merck, Sigma Aldrich, Rehovot, Israel), and volume completed to 20 mL with PBS), incubated at 37 °C for 40 min, washed with ice-cold PBS and then centrifuged at 1500 rpm, for 5 min. The cell pellets were suspended in 500 μL PBS and analyzed using NovoExpress software v1.5.0.

### 4.10. Western Blot Assay

HCC38 and MCF-7 cells (2 × 10^6^) were seeded in complete medium, in T-25 flasks (in triplicates), and incubated at 37 °C, in 5% CO_2_, for 24 h. Cells were then washed with PBS to remove the floating dead cells, and the medium was replaced with serum-free medium. Ep-EVs (1:20 or 1:80) were then added to each flask, while PBS (Cat. No. 02-023-1A; Sartorius, Beit Haemek, Israel) was added to the untreated control cells. After 2 h of incubation, 1.5 mL serum-free medium was added to each flask, and cells were further incubated for 48 h, at 37 °C, in 5% CO_2_. Cells were then washed with PBS to remove floating dead cells. Cells were then harvested, washed with cold PBS and centrifuged at 600× *g* for 5 min. Pellets were lysed with 1X cold RIPA lysis buffer (Cat. No. R0278; Merck, Sigma Aldrich, Rehovot, Israel) supplemented with protease inhibitors (Cat. No. PPC2020; Merck, Sigma Aldrich, Rehovot, Israel) and then vigorously vortexed, placed on ice for 30 min, and then centrifuged at 14,000× *g* for 20 min. The supernatant was transferred to a new tube, and protein concentration was determined using the Pierce BCA Protein Assay (Cat. No. 23225; Rhenium, Modiin, Israel). Cell lysates (40 μg protein) were mixed with 15 μL sample buffer (Cat. No. 70607-3; IMBH, IM Beit HaEmek, Israel) and 2 μL β-mercaptoethanol (Cat. No. 444203; Merck, Sigma Aldrich, Rehovot, Israel), boiled at 95 °C, for 5 min, placed on ice for 1 min, spun down at 16,000× *g* for 1 min, and then resolved by 14% polyacrylamide gel electrophoresis (PAGE). Proteins were then transferred from the gel to a PVDF (polyvinylidene difluoride) membrane using a dedicated BioRad apparatus (Merck, Sigma Aldrich, Rehovot, Israel). Thereafter, the membrane was blocked with 10% bovine serum albumin (Cat. No. NC0131BA; Rhenium, Modiin, Israel), for 1 h, and then immunoblotted with primary antibodies specific for p53 (DO-1) (Cat. No. sc-126, Santa Cruz Biotechnology;, Shoham, Israel), and later with primary antibodies specific for β-actin (Cat. No. sc-47778, Santa Cruz Biotechnoloy, Shoham, Israel), overnight at 4 °C. After washing the membrane three times with TTBS buffer (Cat. No. 002089232300, Merck, Sigma Aldrich, Rehovot, Israel) for 5 min each, it was incubated for 1 h, at room temperature, with relevant HRP-conjugated secondary antibodies (Cat. No. sc-525409, Santa Cruz, Shoham, Israel) diluted in 5% non-fat milk powder dissolved in TTBS buffer. Blot bands were visualized by Prime ECL (Bio-Lab Ltd., Jerusalem, Israel) and imaged using a Bio-Rad ChemicDoc XRS+ system (BioRad Ltd., Rishon LeZion, Israel).

### 4.11. Ep-EV Uptake

HCC38 and MCF-7 cells (1 × 10^5^ cells per slide slot, Cat. PEZGS0416, Sigma Aldrich, Rehovot, Israel) were seeded and cultured overnight at 37 °C, in 5% CO_2_, and then counterstained with Hoechst 33342 nuclear stain (Cat. No. H1399; Invitrogen, Thermo Fisher Scientific, USA) for 15 min, in the dark. Cells were then washed three times with PBS and exposed to labeled Ep-EVs for 18 h, (dilutions of 0 (PBS only), 1:160, 1:80, and 1:20) in triplicate and washed before analysis. Ep-EVs were labeled with lipophilic PKH67 dye, according to the manufacturer’s protocol (Cat. No. 4103975325; Sigma Aldrich, Israel). Briefly, 0.5 mL EVs suspended in PBS was mixed with 2 µL PKH67 in 0.5 mL C diluent and incubated for 5 min before being washed three times and ultracentrifuged. Total uptake of labeled EVs was initially quantified using a Novocyte flow-cytometer, Novoexpress program v1.5.0. (Lumitron ltd., Petah Tikva, Israel, in the Pacific blue and FITC channels. Qualitative uptake of Ep-EVs was also analyzed using an FV4000 confocal microscope (Olympus, Hamburg, Germany).

### 4.12. Statistical Analysis

Statistical analyses were performed using SPSS Statistics software (software version 29), and graphical representations were generated using Microsoft Excel. Data are presented as mean ± standard deviation (SD). The unit of replication (n) represents independent biological replicates, defined as experiments performed on separate occasions using independent cell cultures. For experiments involving multiple treatment dilutions, overall group differences were first assessed using one-way analysis of variance (ANOVA). Planned comparisons between each treatment and the control were performed using two-tailed unpaired Student’s *t*-tests, or Welch’s *t*-test when variances were unequal. When a significant ANOVA result was obtained, post hoc pairwise comparisons were conducted using two tailed Welch’s *t*-tests. Results were considered significant at * *p* < 0.05, ** *p* < 0.01, and *** *p* < 0.005.

## Data Availability

The original contributions presented in this study are included in the article. Further inquiries can be directed to the corresponding author(s).

## References

[1] Bray F., Laversanne M., Sung H., Ferlay J., Siegel R.L., Soerjomataram I., Jemal A. (2024). Global cancer statistics 2022: GLOBOCAN estimates of incidence and mortality worldwide for 36 cancers in 185 countries. CA A Cancer J. Clin..

[2] Saha T., Solomon J., Samson A.O., Gil-Henn H. (2021). Invasion and Metastasis as a Central Hallmark of Breast Cancer. J. Clin. Med..

[3] Siegel R.L., Miller K.D., Jemal A. (2019). Cancer statistics, 2019. CA A Cancer J. Clin..

[4] Ben-Dror J., Shalamov M., Sonnenblick A. (2022). The History of Early Breast Cancer Treatment. Genes.

[5] Sledge G.W., Mamounas E.P., Hortobagyi G.N., Burstein H.J., Goodwin P.J., Wolff A.C. (2014). Past, present, and future challenges in breast cancer treatment. J. Clin. Oncol..

[6] Gustavsson B., Carlsson G., Machover D., Petrelli N., Roth A., Schmoll H.J., Tveit K.M., Gibson F. (2015). A review of the evolution of systemic chemotherapy in the management of colorectal cancer. Clin. Color. Cancer.

[7] Wang H., Mao X. (2020). Evaluation of the Efficacy of Neoadjuvant Chemotherapy for Breast Cancer. Drug Des. Dev. Ther..

[8] Anampa J., Makower D., Sparano J.A. (2015). Progress in adjuvant chemotherapy for breast cancer: An overview. BMC Med..

[9] Wang X., Zhang H., Chen X. (2019). Drug resistance and combating drug resistance in cancer. Cancer Drug Resist..

[10] Xiong X., Zheng L.W., Ding Y., Chen Y.F., Cai Y.W., Wang L.P., Huang L., Liu C.C., Shao Z.M., Yu K.D. (2025). Breast cancer: Pathogenesis and treatments. Signal Transduct. Target. Ther..

[11] Zafar A., Khatoon S., Khan M.J., Abu J., Naeem A. (2025). Advancements and limitations in traditional anti-cancer therapies: A comprehensive review of surgery, chemotherapy, radiation therapy, and hormonal therapy. Discov. Oncol..

[12] Jiang W., Zhong S., Chen Z., Qian J., Huang X., Zhang H., Wen L., Zhang Y., Yao G. (2023). 2D-CuPd nanozyme overcome tamoxifen resistance in breast cancer by regulating the PI3K/AKT/mTOR pathway. Biomaterials.

[13] Burstein H.J., Curigliano G., Thürlimann B., Weber W.P., Poortmans P., Regan M.M., Senn H.J., Winer E.P., Gnant M. (2021). Customizing local and systemic therapies for women with early breast cancer: The St. Gallen International Consensus Guidelines for treatment of early breast cancer 2021. Ann. Oncol..

[14] Swain S.M., Shastry M., Hamilton E. (2023). Targeting HER2-positive breast cancer: Advances and future directions. Nat. Rev. Drug Discov..

[15] Bruni S., Mauro F.L., Proietti C.J., Cordo-Russo R.I., Rivas M.A., Inurrigarro G., Dupont A., Rocha D., Fernández E.A., Deza E.G. (2023). Blocking soluble TNFα sensitizes HER2-positive breast cancer to trastuzumab through MUC4 downregulation and subverts immunosuppression. J. Immunother. Cancer.

[16] Maximiano S., Magalhães P., Guerreiro M.P., Morgado M. (2016). Trastuzumab in the Treatment of Breast Cancer. BioDrugs.

[17] Nahta R., Esteva F.J. (2006). HER2 therapy: Molecular mechanisms of trastuzumab resistance. Breast Cancer Res..

[18] Kadriya A., Falah M. (2023). Nanoscale Phytosomes as an Emerging Modality for Cancer Therapy. Cells.

[19] Rosenberger L., Ezquer M., Lillo-Vera F., Pedraza P.L., Ortúzar M.I., González P.L., Figueroa-Valdés A.I., Cuenca J., Ezquer F., Khoury M. (2019). Stem cell exosomes inhibit angiogenesis and tumor growth of oral squamous cell carcinoma. Sci. Rep..

[20] Sun H., Bhandari K., Burrola S., Wu J., Ding W.Q. (2022). Pancreatic Ductal Cell-Derived Extracellular Vesicles Are Effective Drug Carriers to Enhance Paclitaxel’s Efficacy in Pancreatic Cancer Cells through Clathrin-Mediated Endocytosis. Int. J. Mol. Sci..

[21] Abas B.I., Demirbolat G.M., Cevik O. (2022). Wharton jelly-derived mesenchymal stem cell exosomes induce apoptosis and suppress EMT signaling in cervical cancer cells as an effective drug carrier system of paclitaxel. PLoS ONE.

[22] Reza A., Choi Y.J., Yasuda H., Kim J.H. (2016). Human adipose mesenchymal stem cell-derived exosomal-miRNAs are critical factors for inducing anti-proliferation signalling to A2780 and SKOV-3 ovarian cancer cells. Sci. Rep..

[23] Kalluri R., LeBleu V.S. (2020). The biology, function, and biomedical applications of exosomes. Science.

[24] Zhu L., Kalimuthu S., Gangadaran P., Oh J.M., Lee H.W., Baek S.H., Jeong S.Y., Lee S.W., Lee J., Ahn B.C. (2017). Exosomes Derived From Natural Killer Cells Exert Therapeutic Effect in Melanoma. Theranostics.

[25] Wandrey M., Jablonska J., Stauber R.H., Gül D. (2023). Exosomes in Cancer Progression and Therapy Resistance: Molecular Insights and Therapeutic Opportunities. Life.

[26] Yeung C.H., Wang K., Cooper T.G. (2012). Why are epididymal tumours so rare?. Asian J. Androl..

[27] Breton S., Ruan Y.C., Park Y.J., Kim B. (2016). Regulation of epithelial function, differentiation, and remodeling in the epididymis. Asian J. Androl..

[28] Browne J.A., Leir S.H., Yin S., Harris A. (2019). Transcriptional networks in the human epididymis. Andrology.

[29] Thayer K.M., Carcamo C. (2020). Homologs of the Tumor Suppressor Protein p53: A Bioinformatics Study for Drug Design. MOJ Proteom. Bioinform..

[30] Zhou W., De Iuliis G.N., Dun M.D., Nixon B. (2018). Characteristics of the Epididymal Luminal Environment Responsible for Sperm Maturation and Storage. Front. Endocrinol..

[31] Belleannée C., Calvo É., Caballero J., Sullivan R. (2013). Epididymosomes convey different repertoires of microRNAs throughout the bovine epididymis. Biol. Reprod..

[32] Abe C., Bhaswant M., Miyazawa T., Miyazawa T. (2023). The Potential Use of Exosomes in Anti-Cancer Effect Induced by Polarized Macrophages. Pharmaceutics.

[33] Bae S., Brumbaugh J., Bonavida B. (2018). Exosomes derived from cancerous and non-cancerous cells regulate the anti-tumor response in the tumor microenvironment. Genes Cancer.

[34] Mesdom P., Colle R., Lebigot E., Trabado S., Deflesselle E., Fève B., Becquemont L., Corruble E., Verstuyft C. (2020). Human Dermal Fibroblast: A Promising Cellular Model to Study Biological Mechanisms of Major Depression and Antidepressant Drug Response. Curr. Neuropharmacol..

[35] Gong Y., Kong T., Ren X., Chen J., Lin S., Zhang Y., Li S. (2020). Exosome-mediated apoptosis pathway during WSSV infection in crustacean mud crab. PLoS Pathog..

[36] Kim H.I., Park J., Zhu Y., Wang X., Han Y., Zhang D. (2024). Recent advances in extracellular vesicles for therapeutic cargo delivery. Exp. Mol. Med..

[37] Lau N.C.H., Yam J.W.P. (2023). From Exosome Biogenesis to Absorption: Key Takeaways for Cancer Research. Cancers.

[38] Tripathi S., Sharma Y., Kumar D. (2025). Biological Cargo: Exosomes and their Role in Cancer Progression and Metastasis. Curr. Top. Med. Chem..

[39] Lacroix M., Toillon R.A., Leclercq G. (2006). p53 and breast cancer, an update. Endocr. Relat. Cancer.

[40] Wistuba I.I., Behrens C., Milchgrub S., Syed S., Ahmadian M., Virmani A.K., Kurvari V., Cunningham T.H., Ashfaq R., Minna J.D. (1998). Comparison of features of human breast cancer cell lines and their corresponding tumors. Clin. Cancer Res..

[41] Hessvik N.P., Llorente A. (2018). Current knowledge on exosome biogenesis and release. Cell Mol. Life Sci..

[42] Sidhom K., Obi P.O., Saleem A. (2020). A Review of Exosomal Isolation Methods: Is Size Exclusion Chromatography the Best Option?. Int. J. Mol. Sci..

[43] Zhao Y., Liu T., Zhou M. (2022). Immune-Cell-Derived Exosomes for Cancer Therapy. Mol. Pharm..

[44] Zwaal R.F., Comfurius P., Bevers E.M. (2005). Surface exposure of phosphatidylserine in pathological cells. Cell Mol. Life Sci..

[45] Chaudhri R.A., Hadadi A., Lobachev K.S., Schwartz Z., Boyan B.D. (2014). Estrogen receptor-alpha 36 mediates the anti-apoptotic effect of estradiol in triple negative breast cancer cells via a membrane-associated mechanism. Biochim. Biophys. Acta.

[46] So M.C., Hwang H.P., Lee C.H., Youn H.J., Jung S.H., Kim J.C. (2006). Up-regulation of Pi3k/Akt Signaling by 17β-estradiol through Activation Of Estrogen Receptor-α in Breast Cancer Cells. J. Breast Cancer.

[47] Rezaeian A., Khatami F., Heidari Keshel S., Akbari M.R., Mirzaei A., Gholami K., Mohammadi Farsani R., Aghamir S.M.K. (2022). The effect of mesenchymal stem cells-derived exosomes on the prostate, bladder, and renal cancer cell lines. Sci. Rep..

[48] Anusha R., Ashin M., Priya S. (2023). Ginger exosome-like nanoparticles (GELNs) induced apoptosis, cell cycle arrest, and anti-metastatic effects in triple-negative breast cancer MDA-MB-231 cells. Food Chem. Toxicol..

[49] Raimondo S., Naselli F., Fontana S., Monteleone F., Lo Dico A., Saieva L., Zito G., Flugy A., Manno M., Di Bella M.A. (2015). Citrus limon-derived nanovesicles inhibit cancer cell proliferation and suppress CML xenograft growth by inducing TRAIL-mediated cell death. Oncotarget.

[50] Hashemi Z.S., Ghavami M., Mohammadi F., Shokrollahi Barough M., Shokati F., Asghari S., Khalili S., Akbari Yekta M., Ghavamzadeh A., Sarrami Forooshani R. (2024). Doxorubicin-loaded NK exosomes enable cytotoxicity against triple-negative breast cancer spheroids. Iran. J. Basic Med. Sci..

[51] Christianson H.C., Svensson K.J., van Kuppevelt T.H., Li J.P., Belting M. (2013). Cancer cell exosomes depend on cell-surface heparan sulfate proteoglycans for their internalization and functional activity. Proc. Natl. Acad. Sci. USA.

[52] Hustedt N., Durocher D. (2016). The control of DNA repair by the cell cycle. Nat. Cell Biol..

[53] Lezaja A., Altmeyer M. (2018). Inherited DNA lesions determine G1 duration in the next cell cycle. Cell Cycle.

[54] Long H.-y., Huang Q.-x., Yu Y.-y., Zhang Z.-b., Yao Z.-w., Chen H.-b., Feng J.-w. (2019). Dehydrocostus lactone inhibits in vitro gastrinoma cancer cell growth through apoptosis induction, sub-G1 cell cycle arrest, DNA damage and loss of mitochondrial membrane potential. Arch. Med. Sci..

[55] Wang H.M., Chiu C.C., Wu P.F., Chen C.Y. (2011). Subamolide E from Cinnamomum subavenium induces sub-G1 cell-cycle arrest and caspase-dependent apoptosis and reduces the migration ability of human melanoma cells. J. Agric. Food Chem..

[56] Chaudhry G.E., Md Akim A., Sung Y.Y., Sifzizul T.M.T. (2022). Cancer and apoptosis: The apoptotic activity of plant and marine natural products and their potential as targeted cancer therapeutics. Front. Pharmacol..

[57] Toufektchan E., Toledo F. (2018). The Guardian of the Genome Revisited: P53 Downregulates Genes Required for Telomere Maintenance, DNA Repair, and Centromere Structure. Cancers.

[58] Zhang H., Xu J., Long Y., Maimaitijiang A., Su Z., Li W., Li J. (2024). Unraveling the Guardian: P53’s Multifaceted Role in the DNA Damage Response and Tumor Treatment Strategies. Int. J. Mol. Sci..

[59] Mantovani F., Collavin L., Del Sal G. (2019). Mutant p53 as a guardian of the cancer cell. Cell Death Differ..

[60] Rivlin N., Brosh R., Oren M., Rotter V. (2011). Mutations in the p53 Tumor Suppressor Gene: Important Milestones at the Various Steps of Tumorigenesis. Genes Cancer.

[61] Li X., Niu Z., Sun C., Zhuo S., Yang H., Yang X., Liu Y., Yan C., Li Z., Cao Q. (2022). Regulation of P53 signaling in breast cancer by the E3 ubiquitin ligase RNF187. Cell Death Dis..

[62] Webber J., Clayton A. (2013). How pure are your vesicles?. J. Extracell. Vesicles.

[63] Mulcahy L.A., Pink R.C., Carter D.R. (2014). Routes and mechanisms of extracellular vesicle uptake. J. Extracell. Vesicles.

[64] Welsh J.A., Goberdhan D.C.I., O’Driscoll L., Buzas E.I., Blenkiron C., Bussolati B., Cai H., Di Vizio D., Driedonks T.A.P., Erdbrügger U. (2024). Minimal information for studies of extracellular vesicles (MISEV2023): From basic to advanced approaches. J. Extracell. Vesicles.

[65] Li P., Kaslan M., Lee S.H., Yao J., Gao Z. (2017). Progress in Exosome Isolation Techniques. Theranostics.

[66] Hassellöv M., Readman J.W., Ranville J.F., Tiede K. (2008). Nanoparticle analysis and characterization methodologies in environmental risk assessment of engineered nanoparticles. Ecotoxicology.

